# Nobody Is Perfect: ERP Effects Prior to Performance Errors in Musicians Indicate Fast Monitoring Processes

**DOI:** 10.1371/journal.pone.0005032

**Published:** 2009-04-01

**Authors:** Clemens Maidhof, Martina Rieger, Wolfgang Prinz, Stefan Koelsch

**Affiliations:** 1 Max Planck Institute for Human Cognitive and Brain Sciences, Leipzig, Germany; 2 Department of Psychology, University of Sussex, Brighton, United Kingdom; University of Sydney, Australia

## Abstract

**Background:**

One central question in the context of motor control and action monitoring is at what point in time errors can be detected. Previous electrophysiological studies investigating this issue focused on brain potentials elicited *after* erroneous responses, mainly in simple speeded response tasks. In the present study, we investigated brain potentials before the commission of errors in a natural and complex situation.

**Methodology/Principal Findings:**

Expert pianists bimanually played scales and patterns while the electroencephalogram (EEG) was recorded. Event-related potentials (ERPs) were computed for correct and incorrect performances. Results revealed differences already 100 ms prior to the onset of a note (i.e., prior to auditory feedback). We further observed that erroneous keystrokes were delayed in time and pressed more slowly.

**Conclusions:**

Our data reveal neural mechanisms in musicians that are able to detect errors prior to the execution of erroneous movements. The underlying mechanism probably relies on predictive control processes that compare the predicted outcome of an action with the action goal.

## Introduction

Musical performance is a highly complex and demanding challenge for the human brain [1]–[3]. For example, a pianist playing a Beethoven sonata has to retrieve from memory which notes have to be played, and in which order this has to be done. Then, the corresponding motor programs have to be activated in order to execute the right movements at the right time with the right intensity. Last but not least, the pianist permanently has to monitor and evaluate the effects of the executed actions for correctness. Importantly, all the processes are constantly overlapping in time. Even though the pianist tries to avoid errors like hitting the wrong key, such errors nevertheless occasionally occur. One question that arises in the context of any kind of motor expertise (in our case piano playing) is at what point in time errors are actually detected by the sensorimotor system. More specifically, in the present study we investigated whether errors are detected before a movement is fully executed.

In the motor control literature, it is assumed that fast movement sequences are controlled without external feedback, because the delays of sensory feedback are too long to have an impact on performance (for a review, see [4]). Accordingly, studies in the music domain showed that auditory feedback is not a prerequisite for a successful performance ([5]–[7], for a review, see [8]). These studies found that the complete absence of feedback has mostly no effects on piano performance (whereas specific alterations of auditory feedback can profoundly disrupt performance, see [5]–[7], [9]). Hence, it seems possible that monitoring mechanisms in pianists can operate without auditory feedback, i.e. without the perception of an auditory action-effect.

Furthermore, a behavioral study tried to investigate whether motor experts can detect errors before the movement is completed [10]. That study found that incorrect responses of expert typists were less forceful than correct responses. However, it is not clear whether this effect reflects error-specific processing or results from less activation of the incorrect response (see e.g. [11]). In addition, no real-time correlate of electrical brain activity (e.g., EEG) was recorded. Recording EEG is a technique particularly suited to investigate the time course of cognitive processes on a fine-grained time-scale, as for example the time an error is detected.

EEG-studies on error processing (for reviews, see [12]–[14]) isolated a component of the event-related potential (ERP) appearing shortly *after* participants commit an error in a variety of speeded response tasks (termed the error-related negativity, ERN or Ne [15], [16]). The ERN/Ne typically peaks around 50–100 ms after incorrect responses, regardless of the modality in which the stimulus is presented, and regardless of the modality in which the response is made.

Although the ERN/Ne typically appears after the commission of errors, a recent study [17] found increased negativities before participants committed errors in a speech production task. Participants were presented with sequences of word pairs with identical initial phonemes (e.g., “ball doze”, “bash door”, “bean deck”). Every few trials, a word pair was marked for overt articulation. Importantly, in 10% of the sequences the initial phonemes of the last word pair were exchanged (e.g. “darn bore”). When participants are required to vocalize those last word pairs, they are likely to commit errors (e.g. “barn dore”), because two competing speech plans are activated and interfere with each other. This study [17] found an increased negativity after the presentation of the last word pair, and a second negativity after the presentation of the vocalization prompt. However, it remained unclear when exactly participants started to produce speech, and hence the timing of this error response is not evident. Furthermore, participants saw in each trial the stimuli that induced conflict and hence the speech errors. Therefore, the observed ERP effect might have reflected the resolution of conflict in erroneous trials, rather than the detection of an upcoming error. Thus, neural correlates of error detection prior to error execution have remained elusive.

In the present study we investigated expert pianists performing from memory while we recorded the EEG. That is, we investigated highly trained experts committing errors in a complex situation, in which participants did not react to external conflict-inducing stimuli. We compared the brain potentials before and after correct and incorrect keystrokes. More specifically, we hypothesized that differences in the ERP pattern of correct and incorrect keystrokes would occur even before the completion of the movement.

## Methods

### Participants

Ten highly trained pianists (6 female; mean age 24.3 years, *SD* = 2.8 years) took part in the study. Participants had on average 15.5 years of formal piano training (*SD* = 4.5 years) and were students at the music conservatory in Leipzig. All participants were right-handed according to the Edinburgh Handedness Inventory [18] (mean laterality quotient: 90.5, *SD* = 11.2) and gave informed written consent prior to the experiment. The study was approved by the local ethics committee of the University of Leipzig, and conducted in accordance with the Declaration of Helsinki.

### Material and Apparatus

**Figure 1:**
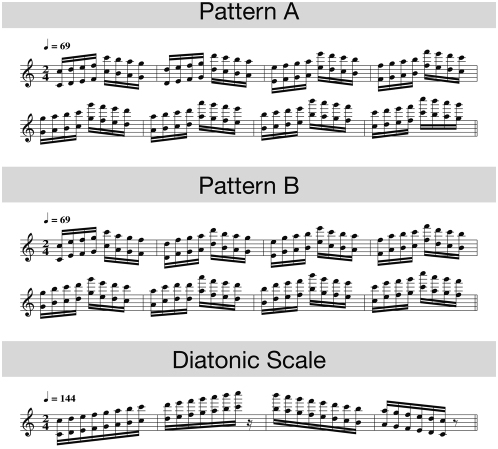
Examples of the stimulus material. A) Pattern A in C-Major. B) Pattern B in C-Major and C) a diatonic scale in C-Major.

The stimuli consisted of major scales and two similar scale-like patterns in two voices (see Figure 1). In each of 24 experimental blocks, the stimuli had to be produced in different major keys in one of the following two orders: C-Major/E-Major/D-Major/F#-Major, or G-Major/B-Major/A-Major (in case of scales, these sequences were repeated). The order of blocks was randomized with the constraints that no identical stimulus type (scale, pattern A, pattern B) occurred in direct succession and that stimuli with the same order of major keys occurred maximally two times in direct succession.

The instructed tempo for the scales was 144 beats per minute (bpm) and for the patterns 69 bpm, i.e. each note event ( = two simultaneous notes) in scales should be produced every 104 ms and in patterns every 217 ms. Randomly between every 40th to 60th produced note, the auditory feedback of a single note was manipulated by lowering the pitch of one note by one semitone. The results of that manipulation will be reported elsewhere.

The pianists performed on a Yamaha digital piano (Clavinova CLP 130), and listened to their performances via AKG 240 studio headphones at comfortable listening levels (approximately 65 dB, dependent on the velocity of a keypress). All tones had the standard MIDI (Musical Instrument Digital Interface) piano timbre generated by a Roland JV-2080 synthesizer (Hamamatsu, Japan).

### Procedure

In the first part of the experiment (ca. 20 min), pianists listened to prerecorded versions of the sequences, which were presented in the same order as the pianists were later required to perform them. Following a practice period with the notation in front of them, participants were blindfolded (to exclude visual feedback and to increase the task difficulty) and instructed to reproduce these stimuli bimanually (parallel in octaves) in the same tempo as they heard them before, i.e. stimuli should be reproduced from memory. If they were not able to perform in the same tempo, they chose their fastest possible tempo. They were informed about the feedback manipulations, and instructed to continue playing, in the event of a feedback manipulation as well as a mistake. When required, participants could rest between two blocks. Before each block, an acoustic instruction was played, informing the participants which scales or patterns they had to produce in the following block. Each performance session lasted approximately 1.5–2 h, and pianists were paid for their participation.

### Data Recording and Analysis

Testing was carried out in an acoustically and electrically shielded EEG cabin. Musical data were processed in MIDI format with a modified version of the open source program “FTAP” [19], [20]. To synchronize musical and electrophysiological data, this program sent trigger signals concurrently with every 5th keypress (and concurrently with the feedback manipulations) to the EEG acquisition computer. For offline analyses, the MIDI information (including timing information, keypress velocities, and pitch) was saved on a hard disk.

The EEG was recorded with 60 Ag/AgCl scalp electrodes placed according to the extended 10–20 system (FP1, FP2, AF7, AF3, AFZ, AF4, AF8, F9, F7, F5, F3, FZ, F4, F6, F8, F10, FT9, FT7, FC5, FC3, FCZ, FC4, FC6, FT8, FT10, A1, T7, C5, C3, CZ, C4, C6, T8, A2, TP9, TP7, CP5, CP3, CPZ, CP4, CP6, TP8, TP10, P9, P7, P5, P3, PZ, P4, P6, P8, P10, PO7, PO3, POZ, PO4, PO8, O1, OZ, O2), referenced to the electrode at the left mastoid. The ground electrode was placed on the sternum. The horizontal electrooculogram (EOGH) was recorded bipolarly from electrodes placed on the outer left and right canthus and the vertical EOG (EOGV) from electrodes placed below and above the left eye. Impedance was kept below 5 kΩ. EEG signals were digitized with a sampling frequency of 500 Hz.

After data acquisition, EEG data were downsampled to 250 Hz to reduce the data size and re-referenced to the arithmetical mean of both mastoid electrodes. We then performed an independent component analysis (ICA) with standard parameters for artifact removal as implemented in EEGLAB 4.51 (Swartz Center for Computational Neurosciences, La Jolla, CA; http://www.sccn.ucsd.edu/eeglab [21]). After calculating the independent components, artifactual components due to eye movements and blinks were selected based on the following criteria: a component was considered to be artifactual if its topography showed peak activity only over the horizontal or vertical eye electrodes, if it showed a smoothly decreasing power spectrum (which is typical for eye movement artifacts, see [21]), and if the component's activity contributed mainly to the raw EEG signal recorded by the horizontal and vertical eye electrodes. The artifactual components were subtracted from the EEG data, and then the EEG data were filtered with a 0.25–25 Hz bandpass, finite impulse response filter. Subsequently, an automatic rejection procedure was applied: Eye artifacts (which could have still been present after the ICA rejection procedure) were rejected whenever the standard deviation within a 200 ms window centered around each sampling point exceeded 25 µV in the EOG. Artifacts caused by drifts and body movements were eliminated by rejecting sampling points whenever the standard deviation exceeded 25 µV at any electrode either within a 200, or within a 800 ms gliding window.

Performance errors were defined as playing an incorrect key with one hand while pressing the correct key with the other hand. Errors were manually identified off-line. Epochs containing other types of errors like omissions or incorrect keypresses with both hands simultaneously were discarded (on average, there were only 18 trials per participant containing the latter type of error). Only errors that were preceded by a 1 s period of error-free performance (and free of feedback manipulations) were analyzed. Errors were identified separately for the scales and the patterns to take into consideration that the different tempi of both types of stimuli possibly influenced ERP effects. On average, there were only 9 error trials during the performance of the scales, which is insufficient to obtain a reasonable signal-to-noise ratio. Therefore, these data were discarded and we will thus only report the data of the performances of the patterns.

Subsequent to the rejection and filtering procedures, event-related potentials were computed for incorrect (*M* = 62, *SD* = 37) and correct (*M* = 682, *SD* = 187) keypresses for 2000 ms time-locked to the onset of the tones (1000 ms before the onset and 1000 ms after the onset). The baseline was set from 1000 ms to 800 ms before the onset of the tone. For the computation of the signal-to-noise ratio (SNR), we estimated the signal power by determining the highest amplitude in the ERPs between -800 ms and +1000 ms. The noise power was estimated by the standard deviation in the baseline time interval, i.e. between −1000 and −800 ms. The SNR averaged across all participants was 11.1 (*SD* = 5.2).

For statistical analysis, mean ERP amplitude values were calculated for two regions of interest (ROIs) over the midline of the scalp: one anterior with electrodes AFZ, FZ, FCZ, and CZ, and one posterior with electrodes CPZ, PZ, POZ, and OZ. ERPs were statistically analyzed by repeated measures analyses of variance (ANOVAs) with the factors Keypress (correct, incorrect) and AntPos (anterior, posterior). Time windows for statistical analyses of ERP data were chosen based on visual inspection of the grand average and centered around the maximum of the differences between correct and incorrect performed notes. The resulting time windows were −150 to −80 ms (i.e. before the note onset) and 240 to 320 ms (after note onset).

For the behavioral data, we analyzed the MIDI velocities (i.e., the speed at which pianists pressed a key, measured on a scale ranging from 0 to 127; MIDI velocity corresponds to the loudness of the produced tone) of incorrect notes, simultaneous correct notes (played by the other hand), and correct notes when there was no error in either hand. The inter-onset intervals (IOIs) were calculated between the onset of an erroneous note and the onset of the previous note (played by the same hand), between the onset of the simultaneously played correct note and the previous correct note (played by the same hand), and between the onset of successive correct notes (i.e. when there was no error in either hand). Whenever the IOI exceeded 1000 ms, this IOI was discarded. The (signed values of the) asynchronies of keypresses were calculated between errors and the simultaneous correct notes, and between two simultaneous correct notes. All behavioral data were statistically analyzed using repeated measures ANOVAs and paired samples *t*-tests.

## Results

### Behavioral Results

Pianists pressed incorrect and correct keys with different MIDI velocities. An ANOVA with factor condition (incorrect keypress, simultaneous correct keypress, correct keypress when no error was present) showed a significant main effect of condition (*F*(2,18) = 15.18, *p*<.0001). Contrasts indicated that participants pressed incorrect keys with a lower velocity (*M* = 59, *SD* = 8) than the simultaneous correct keypresses (*M* = 63, *SD* = 7; *p* = .003) and keypresses when there was no error present (*M* = 64, *SD* = 7, *p*<.0001). There was no difference between simultaneous correct keypresses (when an error was present in the other hand) and keypresses when there was no error present (*p* = .4). This pattern of results indicates that the lower velocity of the erroneous keypress did not influence the simultaneous correct keypress of the other hand.

Pianists produced correct and incorrect keypresses with different IOIs. An ANOVA with factor condition (IOIs between incorrect keypress and the previous keypress, IOIs between simultaneous correct keypress and the previous correct keypress, IOIs between two successive correct keypresses) showed a main effect of condition (*F*(2,18) = 21.22, *p* = .001). Contrasts revealed that there was no difference between IOIs between incorrect keypress and the previous keypress by the same hand (*M* = 407 ms, *SD* = 106 ms) and IOIs between the simultaneous correct keypress and the previous correct keypress by the same hand (*M* = 404 ms, *SD* = 109 ms; *p* = .24). However, IOIs between incorrect keypress and the previous keypress were prolonged compared to the IOIs between successive correct keypresses when there was no error present (*M* = 367 ms, *SD* = 89 ms; *p* = .001), indicating that the upcoming error slowed down the keypresses (pre-error slowing). Note that the overall tempo (i.e., the IOIs between correct notes) is slower than initially instructed. This is based on the fact that participants could choose their own (fastest possible) tempo whenever they were not able to perform in the instructed tempo, resulting in a slower mean performance speed.

The asynchronies between two simultaneous correct notes (*M* = −2 ms, *SD* = 5 ms) and between an incorrect and a simultaneous correct note (*M* = −4 ms, *SD* = 9 ms) did not significantly differ from each other (*t*(9) = −.71, *p* = .5).

### ERP Results

**Figure 2:**
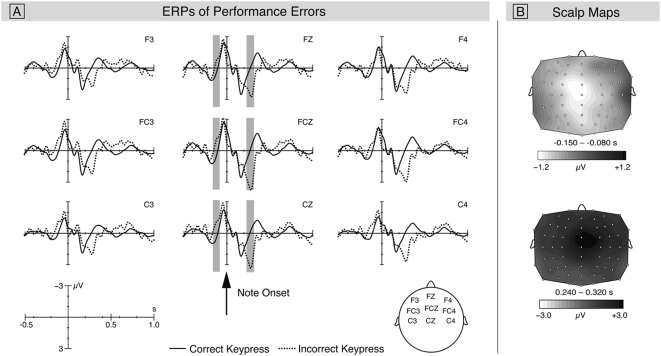
ERP results and scalp distributions of correct and incorrect piano performances. A) Grand-average ERPs elicited by correctly and incorrectly performed keypresses. The arrow indicates the note onset and thus the onset of the auditory feedback. The grey areas show the time windows chosen for statistical analyses for electrodes that were included in the ROIs. Analysis revealed an early increased negative potential prior to the onset of the note (termed pre-error negativity) and a subsequent positive deflection, resembling the early Error positivity (Pe) or the P3a. B) shows the scalp distributions for the difference potentials for correct keypresses subtracted from incorrect keypresses.

Figure 2.A shows the grand-averaged waveforms time-locked to the onset of keypresses. Compared to correct keypresses, incorrect keypresses elicited an increased negativity before a wrong key was actually pressed down. The difference was maximal around 100 ms before the onset of the keypresses and showed a central distribution (see Figure 2.B). An ANOVA for a time window ranging from −150 to −80 ms (i.e., before note onset) with factors Keypress and AntPos indicated a significant main effect of Note (*F*(1,9) = 8.3, *p* = .018), but no interaction between Keypress and AntPos (*F*<1). The pre-error negativity was followed by a later positive deflection with an amplitude maximum at around 280 ms after the onset of an incorrect note. This potential showed a fronto-central scalp topography (see Figure 2.A and 2.B). An ANOVA for a time window from 240 ms to 320 ms with factors Keypress and AntPos revealed a main effect of Keypress (*F*(1,9) = 9.14, *p* = .014) and an interaction between factors Keypress and AntPos (*F*(1,9) = 6.8, *p* = .028), indicating that amplitude values were larger over frontal leads than over parietal leads.

Note that IOIs were prolonged before incorrect keypresses and that incorrect keys were pressed with lower velocities. Hence, the ERP difference occurring before the keypress might be due to motor-related processes, such as adjusting the force of the muscles involved in the movement, rather than cognitive processes underlying error monitoring. Such motor-related processes are expected to be lateralized [22], [23], whereas cognitive processes of error processing do not show hemispheric differences (for reviews, see [12]–[14]). To dissociate between a motor and a cognitive explanation, we tested the lateralization of the ERP difference between correct and incorrect keypresses: The ERPs were analyzed separately for left-hand and right-hand errors, with the assumption that motor-related processes of left-hand errors would be reflected in potentials over right-hemispheric motor areas, and vice versa.

**Figure 3:**
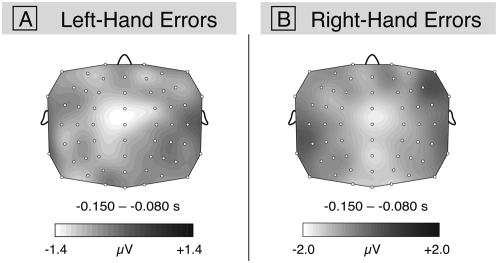
Scalp maps of the difference potentials of left and right-hand errors. A) shows the difference potential for correct keypresses subtracted from left-hand errors and B) the difference potential for correct keypresses subtracted from right-hand errors. Correct keypresses are averaged across both hands.

Potential maps of ERPs of left-hand errors compared to correct notes (averaged across both hands) are shown in Figure 3.A (difference potential: correct notes subtracted from left-hand errors). The analogous comparison for the right-hand errors is shown in Figure 3.B (correct notes subtracted from right-hand errors). For this analysis, three participants were excluded due to the small number of trials (<10). An ANOVA performed on these difference potentials with factors Hand (left, right), and Hemisphere (left ROI including FC3, FC5, C3, and C5 vs. right ROI including FC4, FC6, C4, and C6) showed no effect of Hand (*F*(1,6)<1, *p* = .78), reflecting that the amplitude of ERP effects did not differ between left- and right-hand errors, and no interaction between factors Hand and Hemisphere (*F*(1,6)<1, *p* = .88), reflecting that potentials elicited by the errors were not lateralized.

## Discussion

Brain potentials elicited by correct and incorrect keypresses of expert pianists differed already 100 ms before keypresses were fully executed, and thus prior to the onsets of erroneous tones (pre-error negativity). The early detection of errors is also observable at the behavioral level: IOIs before erroneous keypresses were prolonged, and erroneous keypresses were executed more slowly. However, the asynchronies between the hands did not increase in erroneous trials. 280 ms after erroneous keypresses a frontocentral positive potential was observed. In the following we will first discuss processes occurring before errors are committed and then turn to the processes occurring after errors are committed.

We assume that the ERPs elicited by incorrect performances reflect neural mechanisms that detect errors before they are actually committed, and before auditory feedback is available. Given the speed of movement sequences in the present study (about 3 keypresses with each hand per second), we suggest that internal forward models predicting the sensory consequences of actions [24]–[28] are the basis for detecting the errors even before they were fully executed: Monitoring of fast movements, whose control cannot wait for sensory feedback, has to rely mainly on predictive (feedforward) mechanisms that compare internal action goals with the predicted consequences of planned movements.

Studies investigating the activity of neurons in the primary motor cortex (M1) of non-human primates showed that the latency between the first activity in M1 and movement onset is variable and can range up to several hundred milliseconds [29]–[32], but the typical assumed latency is around 100 to 150 ms (e.g. [33]). At the same time as the motor command is sent from M1 to the periphery, an efference copy (or corollary discharge) is created in brain structures also involved in the generation of the movement. The efference copy is, however, not used to generate the ongoing motor activity, but can be used to predict the outcome of the motor command [24]–[28] (information of efference copies interact at several levels of the central nervous system, and often modulate sensory processing; for reviews, see [34], [35]). The predicted outcome can be compared to the intended outcome, and an error signal is generated whenever there is a mismatch between intended goal and predicted consequence. The error signal can, in turn, modulate the motor command [27].

Accordingly, we assume that the mismatch between the predicted consequence of a planned keypress and the associated internal action goal, as detected by a feedforward control mechanism, is reflected in the pre-error negativity. From the present data we cannot conclude during which part of the movement (planning, initiation, early stages of execution) this feedforward control mechanism exactly occurs. However, it is important to note that a detection mechanism seems to operate before the pianists receive auditory feedback of their errors, i.e. before pianists perceive the auditory results of their actions.

The modulation of the motor command by the error signal of the feedforward mechanism might have resulted in the prolonged IOIs before and the slower velocities of incorrect keypresses, probably reflecting an attempt to avoid the error. In contrast to what one might have assumed, IOIs were not only prolonged for the hand that pressed the incorrect key, but IOIs were also prolonged for the other hand that pressed simultaneously the correct key. This is presumably due to bimanual coupling: studies show that bimanual movements begin and end at similar times, even when they have different parameters (e.g. amplitudes) and movement times differ when the respective movements are performed in isolation by one hand [36]–[38]. Our task required tight bimanual coupling of the hands in terms of the timing. Correspondingly, asynchronies between the hands did not differ when an error was present or not.

One could argue that the pre-error negativity might reflect an error during memory retrieval and, thus, an even earlier stage than motor control or error monitoring. It is assumed that serial-ordering errors (i.e. notes that are intended at another location in the sequence) reflect the current activation of this erroneous element in memory [39], [40]. However, because pianists in our study performed the same tones in parallel with both hands (one octave apart), errors reflecting false memory retrieval should occur in both hands, instead of only in one. Because we only analyzed errors committed by one hand, it is unlikely that the pre-error negativity reflects false retrieval from memory. Moreover, one could also argue that the ERP difference before the note onsets might be due to motor-related processes. Motor execution processes are, however, expected to elicit lateralized EEG potentials [22], [23], which is not consistent with our data: The separate analysis of left-hand and right-hand errors did not reveal any lateralization effect. Therefore, it is unlikely that the ERP difference reflects simply motor-related processes, but rather processes operating at a higher cognitive level, associated with monitoring or control. Finally, one could reason that the increased negativity before incorrectly played notes reflects a process that actually *results* in the production of an error. For instance, a recent study [41] showed that lapses in preparatory attention networks can lead to production errors. In that study the amplitude of the Contingent Negative Variation (CNV), a brain potential indexing preparatory attention, was decreased before stimulus presentation when an erroneous response occurred. Therefore, if lapses in preparatory attention were responsible for the errors in our study, one would have expected a similar decrease in ERP amplitude. However, ERPs elicited before incorrect performances had larger (negative) amplitude values than those elicited before correct performances, rendering such an explanation unlikely. Further, we think that the observed ERP difference in our study occurred too late to reflect lapses in attention. Considering the delay of activity in M1 to movement onset (presumably around 100 to 150 ms), lapses of attention should be observable before that time (as it was reported in [41]), i.e. several hundred milliseconds before the button press. Thus, the fact that an increased negativity (instead of a decreased negative amplitude) was observed, in combination with the observed timing of the effect (around 100 ms before movement completion) renders it improbable that lapses in preparatory attention can account for the present findings. A similar explanation for the present results might be a temporal disengagement of the action monitoring system. Two other studies [42], [43] found that trials preceding erroneous trials (in Eriksen flanker and Stroop tasks) showed an enhanced positivity (compared to trials preceding correct trials), thereby ‘foreshadowing’ errors in future trials. This effect (termed the Error-preceding Positivity, EPP) is thought to reflect “transient deficiencies in the functioning of the monitor system prior to actual execution of an error” [42]. These deficits may be associated with failures to activate adaptive control processes, resulting in occasional future errors. Because we observed no enhanced positivity before production errors, it is unlikely that a disengagement of the action monitoring system is reflected in the observed ERP effect.

The expertise of our participants and the characteristics of our task might explain why we did not observe an ERN (a potential frequently observed following the commission of errors, see [12]–[14] for reviews) or an EPP component: In contrast to most studies investigating error processing (mostly in simple speeded response tasks, including the aforementioned studies [41]–[43]) our participants did not react to external stimuli according to pre-defined arbitrary rules. Instead, they had to select the appropriate motor commands according to internal goals that they formed on the basis of instructions and the musical knowledge stored in their long-term memories. In addition, the present experimental situation reflects a task for which musicians are highly trained, compared to the button press responses to stimuli presented in standard error processing paradigms. Consequently, the error could be detected earlier than in choice reaction tasks. Incorrect notes also violated the regularity of the sequences and thus represented auditory oddballs, which are known to elicit a mismatch negativity (MMN; for a review, see [44]). However, no MMN was visible in the ERPs, perhaps because it was overlapped by the positive potential emerging in a similar latency range (see below). Note that the magnitude of the ERPs (around 3 µV) was rather small compared to the amplitude of ERPs elicited in standard error processing paradigms [12]–[14]. This is probably due to the complexity of our task, involving a range of interacting cognitive processes (e.g., memory retrieval, motor planning, performance monitoring etc., see Introduction). In addition, the simultaneous processing of input from different sensory systems (auditory, tactile, somatosensory) might have influenced the magnitude of the ERPs.

The fronto-central positive potential (emerging around 200 ms and) peaking around 280 ms after incorrect keypresses strongly resembles the Error Positivity (Pe), a potential frequently observed following the ERN in studies of error processing (for reviews, see [45], [46]). Although the functional significance of the Pe has remained rather unclear, three hypotheses about the Pe have emerged: The affective-processing hypothesis [45], [47] suggests that the Pe reflects affective processing of the committed error or its consequences. According to the behavior-adaption hypothesis [48], the Pe reflects the adaptation of response strategy after an error has been perceived, involving remedial performance adjustments following errors. The error-awareness hypothesis [49], [50] proposes that the Pe reflects the conscious recognition of a committed error. There is only little evidence in favor of the first two hypotheses, whereas there are some empirical data supporting the error-awareness hypothesis (e.g. [46], [50], [51]). Another way of addressing the question about the functional significance of this potential is to consider its similarities to the P300 component, which has led to the suggestion that the Pe could reflect a P3b associated with the motivational significance of an error (for a review on the P300, see [52]). The Pe, however, can be decomposed into an early and a late component, very similar to the distinction between P3a (indexing the involuntarily attention switch to novel and deviant stimuli, e.g. [53], [54]) and P3b (taken to reflect memory updating operations after task-relevant stimuli, e.g. [55], [56], but see also [57]). However, there are no studies directly comparing the early Pe with the P3a and the late Pe with the P3b, and therefore it remains unclear whether the early Pe reflects similar processes as the P3a. Based on previous studies [50], [51], [55], [58] we suggest that the positive deflection observed in the present study most likely reflects an early Pe or a P3a. Whether this potential is related to later processing stages of tactile and/or auditory feedback of the error, or simply due to the processing of an oddball stimulus (leading to an involuntary reallocation of attention) remains to be clarified. One way to address this would be to investigate performance errors committed in the absence of auditory feedback: if these errors also elicit the positivity, this potential cannot reflect auditory novelty processing.

In conclusion, the method of investigating motor experts in a natural context, accompanied with on-line measures of electrical brain activity (like EEG), can help to answer crucial questions in the domain of motor control and action monitoring. The occurrence of a pre-error negativity indicates that an early error detection mechanism operates in pianists even before an erroneous movement is fully executed. Our data also show that the early detection of errors influences movement execution, resulting in pre-error slowing of both hands and in keypresses with reduced velocity of the erroneous hand only. We assume that the underlying process is the detection of a mismatch between a predicted sensory consequence of an action and the intended action goal. Thus, our results reveal neural mechanisms that are able to detect errors prior to the execution of erroneous movements.
